# Effect of plasma exchange on COVID-19 associated excess of von Willebrand factor and inflammation in critically ill patients

**DOI:** 10.1038/s41598-022-08853-2

**Published:** 2022-03-21

**Authors:** Felix S. Seibert, Arturo Blazquez-Navarro, Bodo Hölzer, Adrian A. N. Doevelaar, Christian Nusshag, Uta Merle, Christian Morath, Panagiota Zgoura, Rita Dittmer, Sonja Schneppenheim, Jochen Wilhelm, Nina Babel, Ulrich Budde, Timm H. Westhoff

**Affiliations:** 1 Medical Department I, University Hospital Marien Hospital Herne, Ruhr-University Bochum, Hölkeskampring 40, 44625 Herne, Germany; 2 Department of Nephrology, Heidelberg University Hospital, Heidelberg, Germany; 3 Department of Internal Medicine IV, Heidelberg University Hospital, Heidelberg, Germany; 4 Hemostaseology, MEDILYS Laborgesellschaft mbH, Hamburg, Germany

**Keywords:** Viral infection, Nephrology

## Abstract

Ubiquitous microthromboses in the pulmonary vasculature play a crucial role in the pathogenesis of COVID-19 associated acute respiratory distress syndrome (ARDS). Excess of Willebrand factor (vWf) with intravascular multimer formation was identified as a key driver of this finding. Plasma exchange (PLEX) might be a therapeutic option to restore the disbalance between vWf and ADAMTS13. We report the effects of PLEX on vWf, ADAMTS13, inflammatory cytokines and parameters of ventilation. We investigated 25 patients, who were on mechanical ventilation for COVID-19 pneumonia with ARDS at two German university hospitals. All patients received PLEX as an ultima ratio measure for refractory ARDS. VWf antigen (vWf:Ag), ADAMTS13 activity, a cytokine panel mirroring the inflammatory situation and clinical parameters were assessed before and after three to six PLEX therapies with fresh frozen plasma. Before the PLEX sequence there was an excessive release of vWf:Ag (425.4 ± 167.5%) and mildly reduced ADAMTS13 activity (49.7 ± 23.3%). After the PLEX series, there was a significant increase of ADAMTS13 activity to 62.4 ± 17.7% (p = 0.029) and a significant decrease of vWf:Ag to 336.1 ± 138.2% (p = 0.041) resulting in a 63% improvement of the ADAMT13/vWf:Ag ratio from 14.5 ± 10.0 to 23.7 ± 14.6, p = 0.024. Comparison of parameters before and after individual PLEX sessions (n = 35) revealed a mean reduction of vWf from 387.8 ± 165.1 to 213.2 ± 62.3% (p = 0.001) and an increase of ADAMTS13 activity from 60.4 ± 20.1 to 70.5 ± 14.0% (p = 0.001). Parallelly, monocyte chemotactic protein-1 and interleukin-18 decreased significantly (p = 0.034 each). Along the PLEX sequence lactate dehydrogenase (p = 0.001), C-reactive protein (p = 0.001), and positive end expiratory pressure (p = 0.01) significantly decreased accompanied by an improvement of Horovitz index (p = 0.001). PLEX restores the disbalance between ADAMTS13 and vWf:Ag, a driver of immunothrombosis. Moreover, it reduces the inflammatory state and is associated with a benefit of ventilation parameters. These findings render a further rationale to regard PLEX as a therapeutic option in severe COVID-19.

## Introduction

Immunothrombosis is a key feature of Coronavirus disease 2019 (COVID-19) affecting both macro- and microvasculature^1^. Regarding the macrovasculature, COVID-19 patients suffer from arterial and venous thrombembolic events including stroke, limb ischemia, myocardial infarction, deep vein thromboses, and pulmonary embolism^2–4^. Autopsy studies identified pulmonary microvascular thromboses as a major reason for ventilation-perfusion imbalance leading to acute respiratory distress syndrome (ARDS)^5,6^.

A disbalance of von Willebrand factor (vWf) and ADAMTS13 was identified as a main driver of immunothrombosis in COVID-19^7,8^. COVID-19 is associated with ubiquitous endothelial damage leading to excessive release of vWf. ADAMTS13 is a protease that usually cleaves the large string-like molecules of vWf and is necessary to avoid accumulation of vWf multimers in the blood stream. In COVID-19, however, the excessive release of vWf exceeds the capacity of the protease leading to vWf multimers, that act as a matrix for platelet aggregation and thereby cause microvascular thromboses^9^. This mechanism resembles the situation in thrombotic thrombocytopenic purpura (TTP), in which a deficiency of ADAMTS13 causes microangiopathic thromboses. In COVID-19 the ADAMTS13/vWf ratio decreases with the severity of the disease and is a strong and independent predictor of mortality^9^.

The aggregation of platelets at vWf multimers cannot be inhibited by plasmatic anticoagulation, which might explain, why autopsy findings revealed extensive microthromboses in the lungs of COVID-19 patients despite prior plasmatic anticoagulation. Therefore, there is an urgent clinical need for novel preventive and therapeutic strategies to address immunothrombosis in COVID-19. In analogy to TTP, plasma exchange (PLEX) may be considered as a therapeutic option in this context, since it removes vWf and restores ADAMTS13. There are some first preliminary reports on PLEX in COVID-19 intended as a rescue therapy to eliminate proinflammatory cytokines with promising results^10–14^. These studies did not focus, however, on immunothrombosis.

In the present work we present experiences of two German university hospitals, that made use of PLEX as an ultima ratio therapy in COVID-19 associated ARDS. VWf, ADAMTS13, a cytokine panel, and parameters of mechanical ventilation (positive endexpiratory pressure [PEEP] and Horovitz index (partial pressure of oxygen [pO_2_]/fraction of inspired oxygen [FiO_2_]) were assessed before and after three to six sessions of PLEX.

## Results

We enrolled 25 patients with SARS-CoV-2 infection and severe COVID-19 pneumonia with invasive and non-invasive mechanically ventilated ARDS, who underwent three to six sessions of PLEX with human plasma (median n = 5, overall n = 112). Mean age of the COVID-19 population was 67.0 ± 11.9 years. Gender distribution was homogeneous with n = 11 being female (44%). Characteristics of patients are summarized in Table 1. Eight (32%) had severe and 14 (56%) moderate ARDS. 14 (56%) patients died from COVID-19. Mean time of hospitalization was 40.8 ± 24.3 days. 21 patients (84%) were treated with dexamethasone, remdesivir was initiated in 10 (40%) cases (Table 1). Dexamethasone was permanently discontinued before the enrolment in our study in 16% of our patients due to a prior superinfection.

**Table 1:** Population characteristics.

Variables	COVID-19 population (n = 25)
Age	67 ± 11.9
Gender	Female 11 (44%)
	Male 14 (56%)
Initiation of PLEX after admission to the intensive care unit (days)	4 (IQR 3–8)
Median number of PLEX sessions	5 (IQR 4–5)
Dexamethasone	21 (84%)
Remdesivir	10 (40%)
Severe ARDS	8 (32%)
Moderate ARDS	14 (56%)
Mild ARDS	3 (12%)

*IQR* interquartile range, *PLEX* plasmapheresis, *ARDS* acute respiratory distress syndrome.

### Evaluation of parameters before and after the complete PLEX sequence

**Figure 1:**
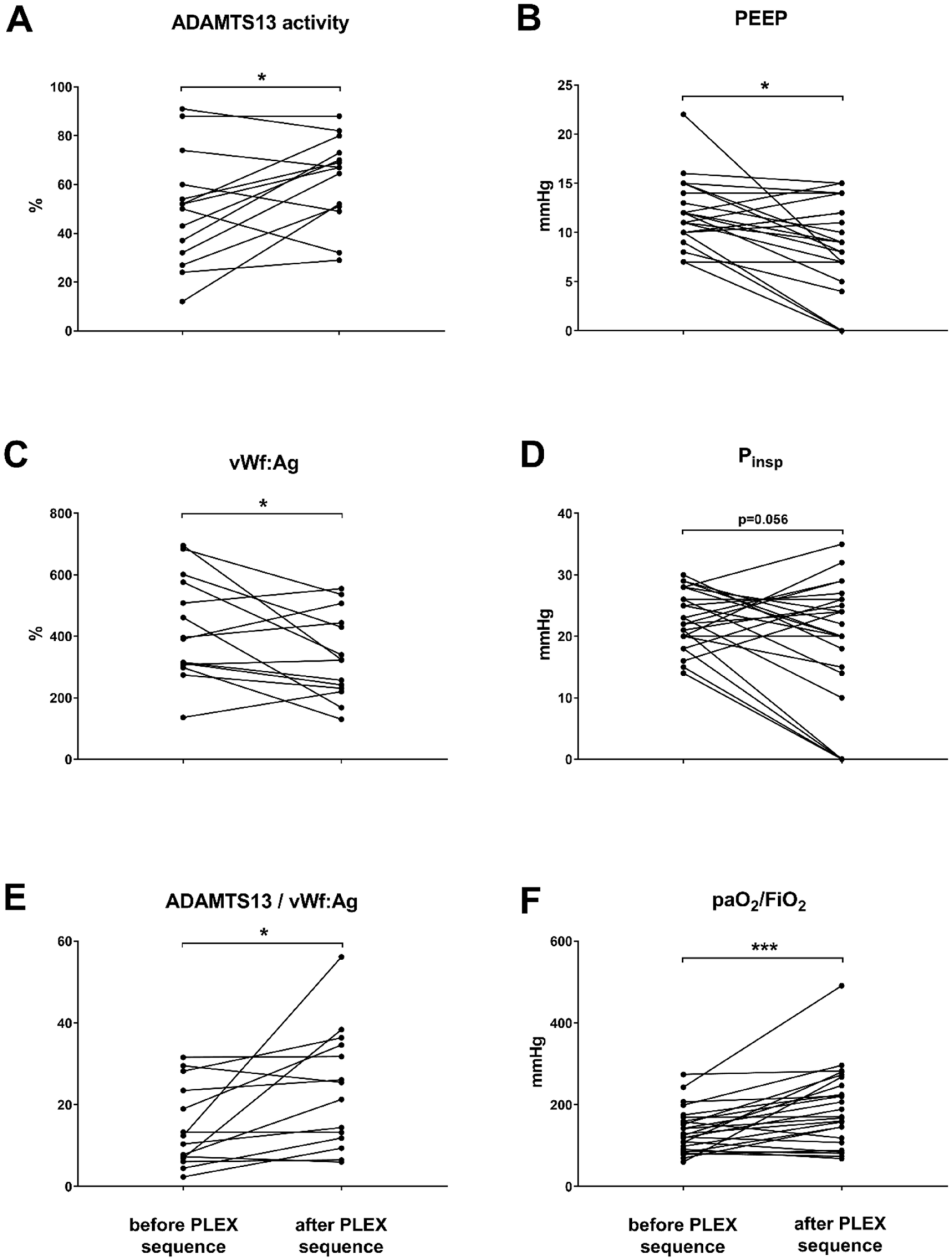
ADAMTS13 and von Willebrand factor (vWf) hemostasis and ventilation parameters before and after a sequence of three to six PLEX sessions. (**A**) ADAMTS13, (**B**) PEEP, (**C**) vWf:Ag, (**D**) P_insp_, and (**E**) ADAMTS13/vWf:Ag, and (**F**) the Horovitz index. Data are analysed by a paired two-tailed t-test. ***p = 0.001, **p < 0.01 and *p < 0.05 were regarded significant. *PEEP* positive endexpiratory pressure, *P*_*insp*_ positive inspiratory pressure, *vWf:Ag* von Willebrand factor antigen.

ADAMTS13 activity was 49.7 ± 23.3% before the PLEX sequence and increased towards 62.4 ± 17.7% (p = 0.029). Parallelly, vWf:Ag decreased from 425.4 ± 167.5 to 336.1 ± 138.2% (p = 0.041). The ADAMTS13/vWf:Ag ratio improved significantly by 63.4% from 14.5 ± 10.0 to 23.7 ± 14.6 (p = 0.024) after completion of the PLEX sequence (Fig. 1, Suppl. Fig 1).

As illustrated in Fig. 1, PEEP was significantly reduced by 26.5% after the PLEX series (11.7 ± 3.2 vs. 8.6 ± 4.7 mmHg, p = 0.009), while P_insp_ tended to decrease (22.9 ± 4.6 vs. 18.1 ± 10.7 mmHg, p = 0.056). The Horovitz index ameliorated significantly by 41.0% from 135.3 ± 54.4 mmHg to 190.8 ± 95.4 mmHg (p = 0.001). We could not observe any significant differences in the need for vasopressors (p = 0.091).

After completion of the PLEX series, lactate dehydrogenase was reduced by 48.3% (608.5 ± 284.7 vs. 314.8 ± 94.6 IU/l, p = 0.001). CRP decreased by 75.2% after the PLEX sequence (16.9 ± 10.8 vs. 4.2 ± 4.7 mg/dl, p = 0.001). Table 2 presents the coagulation parameters of the study population before and after PLEX.

**Table 2:** Clinical parameters and ADAMTS13/vWf balance before and after the complete plasma exchange sequence and individual sessions.

Variables	Complete PLEX sequence (n = 25)			Individual PLEX sessions pre-post comparisons (n = 35)		
	Before	After	p	Before	After	p
ADAMTS13 activity (%)	49.7 ± 23.3	62.4 ± 17.7	**0.029**	60.4 ± 20.1	70.5 ± 14.0	**0.001**
vWf:Ag (%)	425.4 ± 167.5	336.1 ± 138.2	**0.041**	387.8 ± 165.1	213.2 ± 62.3	**0.001**
ADAMTS13/vWf (%)	14.5 ± 10.0	23.7 ± 14.6	**0.024**	19.6 ± 12.5	36.3 ± 14.1	**0.001**
LDH (IU/l)	608.5 ± 284.7	314.8 ± 94.6	**0.001**			
lactate (mmol/l)	1.76 ± 0.55	1.58 ± 0.50	0.234			
CRP (mg/dl)	16.9 ± 10.8	4.2 ± 4.7	**0.001**			
Ferrtin ng/ml	1918 ± 1730	413 ± 294	0.224			
Platelets (/nl)	250.9 ± 85.6	176.0 ± 82.5	**0.001**			
PEEP (mmHg)	11.7 ± 3.2	8.6 ± 4.7	**0.009**			
P_insp_ (mmHg)	22.9 ± 4.6	18.1 ± 10.7	0.056			
Horovitz index (mmHg)	135.3 ± 54.4	190.8 ± 95.4	**0.001**			
Noradrenaline (µg/kg/min)	0.103 ± 0.210	0.037 ± 0.093	0.091			

*PLEX* plasmapheresis, *vWf:Ag* von Willebrand factor antigen, *LDH* lactate dehydrogenase, *CRP* C-reactive protein, *PEEP* positive end-expiratory pressure, *P*_*insp*_ inspiratory pressure.

Significant values are given in bold.

### Evaluation of parameters before and after the individual PLEX sessions

**Figure 2:**
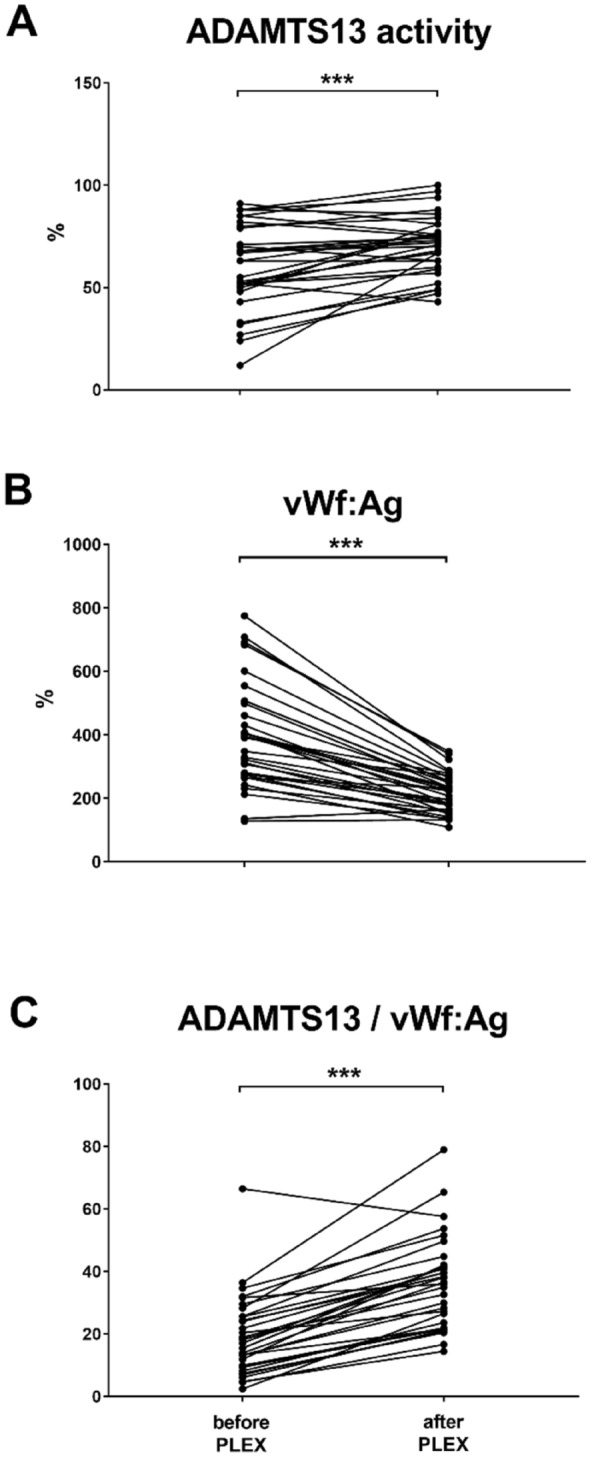
ADAMTS13 and vWf balance before and after individual PLEX sessions. (**A**) ADAMTS13, (**B**) vWf:Ag and (**C**) ADAMTS13/vWf:Ag. Data are analyzed by a paired two-tailed t-test. ***p = 0.001, **p < 0.01 and *p < 0.05 were regarded significant. *vWf:Ag* von Willebrand factor antigen.

**Figure 3:**
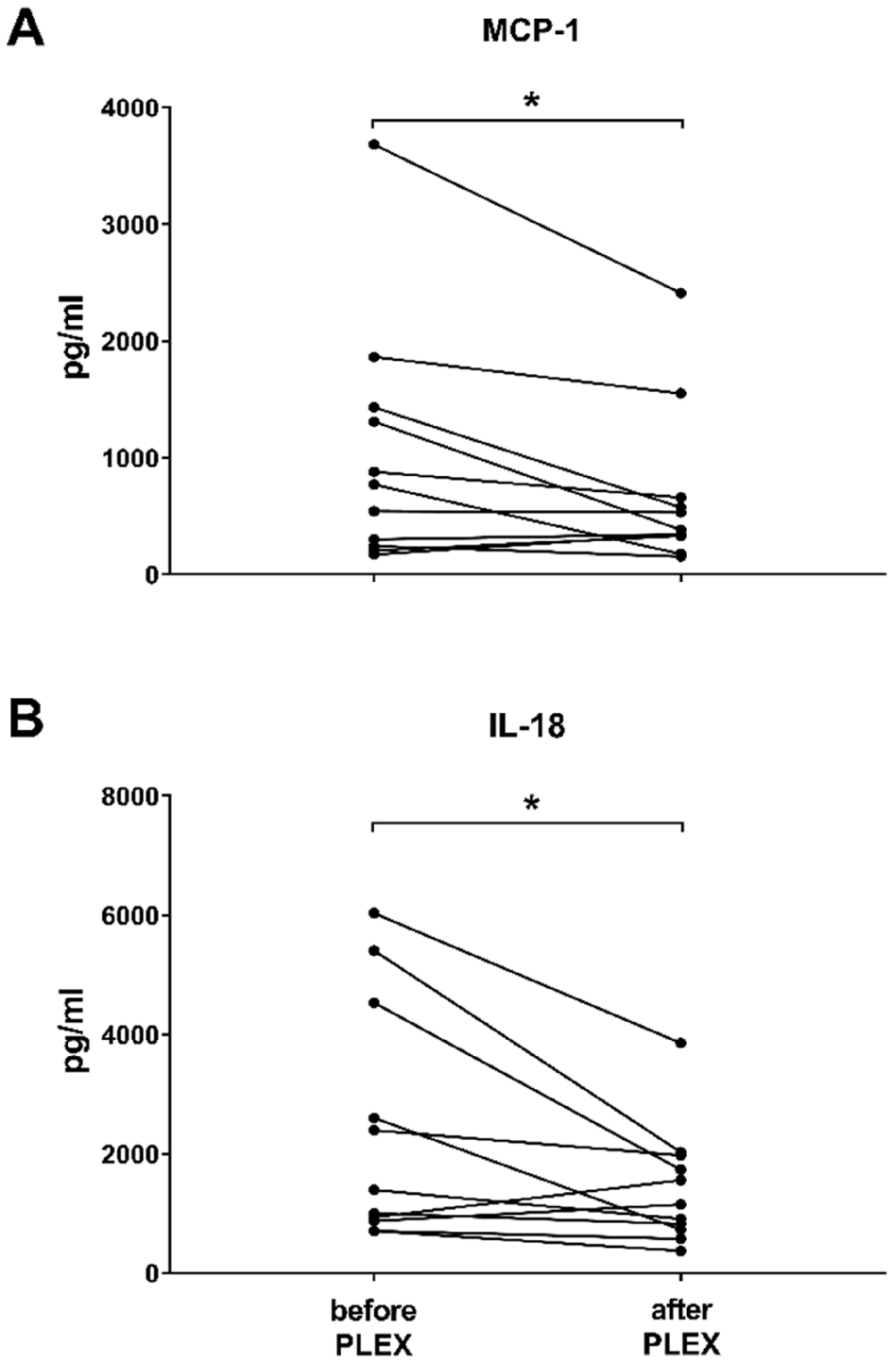
Individual mean concentrations of (**A**) monocyte chemotactic protein-1 (MCP-1) and (**B**) interleukin-18 (IL-18) before and after a singular PLEX session, each point representing one patient. Data before and after treatment are analysed by paired t-test. ***p = 0.001, **p < 0.01 and *p < 0.05 were regarded significant.

Measurements of vWf:Ag and ADAMTS13 were conducted successfully right before and after 35 PLEX sessions (11 patients), as presented in Fig. 2. A PLEX session was associated with a mean reduction by 45.0% of vWf:Ag concentrations, from 387.8 ± 165.1 to 213.2 ± 62.3% (p = 0.001), while ADAMTS13 activity significantly increased by 16.7%, from 60.4 ± 20.1 to 70.5 ± 14.0%, p = 0.001). The ADAMTS13/vWf:Ag ratio was substantially lower before PLEX treatment (19.6 ± 12.5) and showed a significant increase to 36.3 ± 14.1 (p = 0.001; Fig. 2, Table 2). PLEX was associated with significant reductions of two cytokines of the panel described above: monocyte chemotactic protein-1 and interleukin-18 (p = 0.034 each; Fig. 3).

## Discussion

The present retrospective analysis shows, that PLEX is able to reduce the excess of vWf, to increase ADAMTS13 activity, to readjust the ADAMTS13/vWf:Ag ratio and thereby to reduce the risk of immunothrombosis in COVID-19. These effects were accompanied by a reduction of proinflammatory cytokines and an improvement in parameters of mechanical ventilation. The restoration of vWf and ADAMTS13 hemostasis constitutes a novel rationale to regard PLEX as a therapeutic option in SARS-CoV-2 induced ARDS beyond the reduction of the inflammatory milieu. In both centers, PLEX was used as an ultima ratio therapy. Future studies will have to define the optimal timepoint for initiation of PLEX. Randomized trials are underway.

In our previous study, we identified TTP-like vWf multimer patterns as a driver of immunothrombosis in COVID-19^9^. ADAMTS13 is unable to sufficiently abolish the excessive vWf multimer formation. The endothelium plays a crucial role in hemostasis, regulating not only oxidative stress, and permeability but complement activation and pro- and anticoagulant factors as well. Severe COVID-19 is associated with ubiquitous endotheliitis, triggered by the primary SARS-CoV 2 infection and secondarily via a microvascular inflammatory process^15,16^. Moreover, an in vitro study demonstrated that SARS-CoV-2 spike protein can activate the alternative complement pathway^5,17^. The disbalance between ADAMTS13 and vWf represents a further crucial pathomechanism of COVID-19 associated immunothrombosis.

PLEX constitutes a central therapeutic measure in the management of TTP. The goal is not only to remove ADAMTS13 inhibitors, but also to restore the patients’ serum with a sufficient amount of ADAMTS13. In COVID-19, one major effect is the removal of vWf. Noteworthy, however, there is first evidence on a generation of ADAMTS-13 autoantibodies as well^18^. Thus, PLEX restores vWf and ADAMTS13 hemostasis by means of both removal of vWf, delivery of ADAMTS13, and—potentially—elimination of autoantibodies to ADAMTS-13. These mechanisms likely reduce SARS-CoV-2 associated microthrombosis and improve organ perfusion.

With regard to individuals refusing vaccination and those with vaccination breakthrough, the ongoing pandemic constitutes an urgent need for either new therapeutic approaches or those that are already approved for other indications. In the latter context, PLEX was used in order to attenuate circulating cytokines and inflammatory mediators in critically ill patients with COVID-19 as described by small case series with promising results^14,19^. In these reports PLEX was associated with higher extubation rates, and lower 14 days and 28 days all-cause mortality^20^. With regard to the restoration of the physiological vWf/ADAMTS13 balance, our analysis identifies an additional novel explanation for these clinical benefits. The excess of vWf was immediately dampened even by a single session of PLEX. Within the next days, vWf tended to increase again, thus making a sequence of sessions with intervals of 1 or 2 days reasonable. Accordingly, vWf was lower and ADAMTS13 higher at the end of the sequence compared to baseline values. Our cytokine analysis confirms the antiinflammatory potential of PLEX as demonstrated in previous studies^14,21^. PLEX reduced MCP-1 and IL-18 concentrations in the individual sessions. A decrease of thrombocytes cannot clearly be explained. However, platelet consumption is regularly seen on patients undergoing extracorporeal blood treatment and/or treated on the intensive care unit^22^. Due to the amelioration of the thrombotic microangiopathy parameters along the PLEX sequence, this reduction in platelet count cannot be explained through a relative ADAMTS13 deficiency.

Respiratory and ventilatory insufficiency in SARS-CoV-2 associated ARDS results from two major causes: First, alveolar inflammation and second, microthromboses in the pulmonary circulation. Whereas current treatment strategies like dexamethasone or tocilizumab are intended to address excessive inflammation, there is no specific therapeutic option to reduce immunothrombosis so far. The use of plasmatic anticoagulants in therapeutic doses resulted in unacceptable rates of bleeding events and is not recommended for COVID-19 necessitating intensive care medicine as seen in trials like REMAP-CAP, ATTACC and ACTIV-4^23^. PLEX might be a helpful therapeutic adjunct in this context. The extensive microvascular thromboses in the lungs of deceased COVID-19 patients—despite prior low dose or therapeutic anticoagulation—illustrate the need for new therapies to regenerate diffusion capacity and oxygenation. In line with this hypothesis the ventilatory situation was improved in our population as reflected by PEEP and Horovitz index.

In the present study, PLEX served as a rescue therapy. Different kinds of immunomodulatory drug based therapies (e.g. JAK-inhibition) showed favourable effects, but still none of them are seen as an evidence-based ultima ratio therapy for COVID-19. Furthermore, inhaled nitric oxide could provide immediate help and a delay in respiratory deterioration in COVID-19-induced moderate to severe ARDS. However the data appears conflicting, and reports only show a temporary amelioration^24,25^. Lung transplantation constitutes an ultimate rescue therapy of COVID-19 induced lung injury. Obviously, only a few carefully selected patients will be suitable for such therapy. In a letter to the Editor, the authors report a similar survival rate compared to lung transplant recipients, suffering from a different underlying pulmonary disease^26^. The long term outcome, especially with regard to an ongoing pandemic and immunosuppression, remains elusive, but lung transplantation for COVID-19 will not be suitable on a large scale.

The present analysis is limited by its sample size and its retrospective character. PLEX was used in Bochum as an ultima ratio therapy, when there were no other evidence-based ultima ratio therapies available. Due to the continuously evolving data on the management of COVID-19, e.g. concerning anticoagulation and the use of immunosuppressants like tocilizumab, it was not possible to define a sufficiently matching control group. Data about the course of the investigated parameters before and after the individual PLEX sessions, however, allow a precise determination of the PLEX-induced effects.

In conclusion, the present analysis identifies restoration of the SARS-CoV-2 associated physiological balance of vWf and ADAMTS13 as a new rationale to consider PLEX as a therapeutic option in COVID-19. Moreover, it confirms previous findings on a reduction of inflammation and an improvement of ventilation parameters.

## Methods

### Patients

We analysed 25 patients from two university hospital intensive care units, who were tested positive for SARS-CoV-2 by real-time polymerase chain reaction (RT-PCR) analysis in respiratory tract specimen (nasopharyngeal swab test or bronchoalveolar lavage). Patients were recruited at the Ruhr-University Bochum (n = 19) and the Ruprecht-Karls University of Heidelberg (n = 6) in Germany. All patients suffered from a critical course of disease, as categorized by the Robert Koch Institute, including invasive and non-invasive mechanical ventilation and fulfilling parameters for the diagnosis of an acute respiratory distress syndrome^27^. Changes in vWf, ADAMTS13, cytokines, and key parameters of ventilation were defined as clinical endpoints. Retrieval of blood samples was performed as part of an investigation of immunological, inflammatory, and hemostaseological parameters in COVID-19 at Ruhr-University Bochum and in the context of a prospective register study at Heidelberg University Hospital. Informed consent was obtained from all subjects or their legal guardian. Both investigations were approved by the respective ethical committee of Ruhr-University Bochum (20-6886) and Ruprecht-Karls University Heidelberg (S-148/2020).

### Clinical and biochemical parameters

PEEP, inspiratory pressure (P_insp_), and Horovitz index (partial pressure of oxygen [pO_2_]/fraction of inspired oxygen [FiO_2_]) were defined as key ventilatory parameters. Additionally, the need of vasopressors (noradrenaline) was documented. VWf, ADAMTS13, lactate dehydrogenase and platelets were assessed as markers of thrombotic microangiopathy. C-reactive protein and a cytokine panel were used as markers of inflammation: The LEGENDplex Human Inflammation Panel 1 (13-plex) (BioLegend, CA, USA) containing interleukin (IL)-1β, interferon (IFN)-α2, IFN-γ, tumor necrosis factor (TNF)-α, monocyte chemoattractant protein (MCP)-1 (CCL2), IL-6, IL-8, IL-10, IL-12p70, IL-17A, IL-18, IL-23, and IL-33 was used according to the manufacturer’s instruction. The concentration of the analytes was calculated using the LEGENDplex Cloud-based Data Analysis Software v.2021.07.01.

### Plasma exchange (PLEX)

In both centers PLEX was clinically indicated as an ultima ratio therapy of COVID-19 associated ARDS with a Horovitz Quotient ≤ 300 mmHg. Patients were checked for remediable conditions like volume overload or superinfection. Thus, volume status was assessed clinically and via ultrasound and was excluded before therapy induction. The PLEX regimen consisted of three to six sessions with a turnover of one individualised plasma volume. In case of acute clinical amelioration during treatment (clear increase of Horovitz index), or for restricted capacity reasons (e.g. shortage of staff during critical phases of the pandemic), this kind of treatment was individually reconsidered and eventually discontinued. PLEX were performed on the Octo Nova (Diamed, Köln, Germany) with heparin as anticoagulation. Plasma separation was provided via Plasma Flux P2 Dry (Fresenius Medical Care, Germany) or using a Comtech centrifuge (Fresenius Medical Care AG & Co. KGaA, Bad Homburg, Germany). PLEX sessions had a maximum 48-h interval. In addition to retrieval of blood samples before and after the complete PLEX sequence (overall population), blood samples were obtained before and after individual PLEX sessions in a subset of 11 patients.

### Measurement of ADAMTS13 activity and vWf antigen

VWf:Ag and ADAMTS13 activity were analyzed in all patients prior to the first PLEX and after a sequence of three to six PLEX, noteworthy the second specimen was obtained 24 h after the end of the PLEX series. Additionally, blood samples of 11 patients were obtained immediately prior and after a total of 35 individual PLEX sessions, allowing to assess an extinction rate of vWf:Ag and a restoration rate of ADAMTS13. ADAMTS13 activity (%) was analyzed from citrate-plasma using Technozym ADAMTS13 ELISA (Technoclone, Vienna, Austria)^28^. vWf:Ag (%) was measured using a sandwich ELISA with polyclonal antibodies^29^.

### Statistics

Data were checked for Gaussian distribution by D’Agostino Pearson test and are presented as mean ± standard deviation. Changes in ADAMTS13 activity, vWf:Ag and ADAMTS13/vWf:Ag ratios right before and after PLEX were investigated by paired two-tailed t-test. Moreover, a paired two-tailed t-test was used to analyze the evolvement of clinical parameters (PEEP, P_insp_, Horovitz index, noradrenaline, LDH, CRP, lactate, thrombocytes, cytokines) as well as ADAMTS13 activity, vWf:Ag and ADAMTS13/vWf:Ag ratios before and after the complete sequence of PLEX. p < 0.05 was regarded significant. Statistical analyses were performed using Prism 9 (Graph Pad, San Diego, USA).

### Ethical approval

Both investigations were approved by the respective ethical committee of Ruhr-University Bochum (20-6886) and Ruprecht-Karls University Heidelberg (S-148/2020) and performed in accordance with the relevant guidelines and regulations.

## Supplementary Information

Supplementary Figure 1.
