# Supplementary material for: Effect of plasma exchange on COVID-19 associated excess of von Willebrand factor and inflammation in critically ill patients
Source: Sci Rep. 2022 Mar 21;12:4801. doi: 10.1038/s41598-022-08853-2 (PMC8935881; doi:10.1038/s41598-022-08853-2)

**SUPPLEMENTS**

**Figure legends**

**Figure 1**

Individual ADAMTS13/vWf:Ag concentrations right before initiation of a PLEX unit. There remains a tendency for amelioration (p=0.07; Kruskal-Wallis-test) with an increase of 118% (p=0.008; Wilcoxon-test) when comparing the ADAMTS13/vWF:Ag before the first and before the fifth PLEX. PLEX sessions are numbered on the x-axis. ***p=0.001, **p<0.01 and *p<0.05 were regarded significant. PLEX – plasma exchange. vWf:Ag – von Willebrand factor antigen.

**Figures**

Figure 1


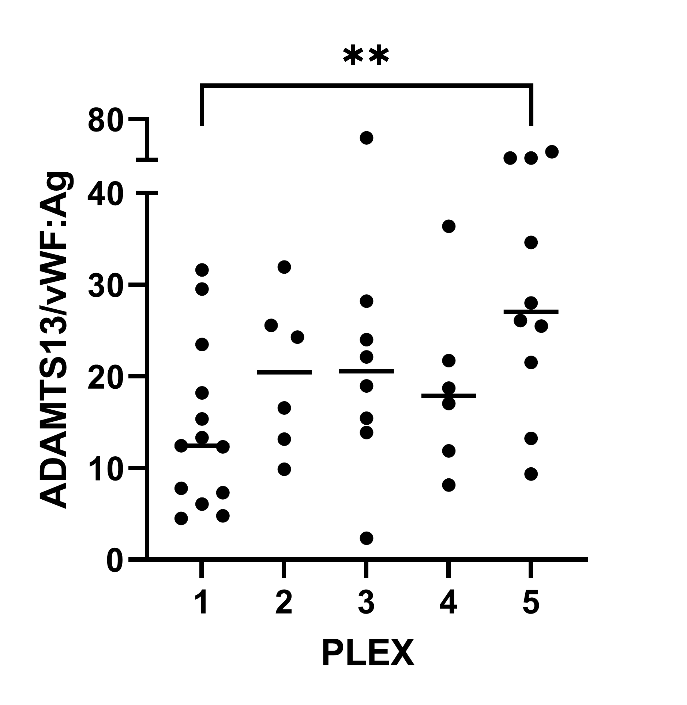

Supplement: Supplementary file 1 — Supplementary Figure 1. [file 41598_2022_8853_MOESM1_ESM.docx]
